# Peptide-membrane interactions of arginine-tryptophan peptides probed using quartz crystal microbalance with dissipation monitoring

**DOI:** 10.1007/s00249-014-0958-9

**Published:** 2014-04-18

**Authors:** Hanna A. Rydberg, Angelika Kunze, Nils Carlsson, Noomi Altgärde, Sofia Svedhem, Bengt Nordén

**Affiliations:** 1 Department of Chemical and Biological Engineering, Chalmers University of Technology, Kemivägen 10, 412 96 Göteborg, Sweden; 2 Department of Applied Physics, Chalmers University of Technology, Fysikgränd 3, 412 96 Göteborg, Sweden

**Keywords:** Cell-penetrating peptides, Antimicrobial peptides, Membrane-active peptides, Peptide-membrane interactions, Quartz crystal microbalance with dissipation monitoring

## Abstract

Membrane-active peptides include peptides that can cross cellular membranes and deliver macromolecular cargo as well as peptides that inhibit bacterial growth. Some of these peptides can act as both transporters and antibacterial agents. It is desirable to combine the knowledge from these two different fields of membrane-active peptides into design of new peptides with tailored actions, as transporters of cargo or as antibacterial substances, targeting specific membranes. We have previously shown that the position of the amino acid tryptophan in the peptide sequence of three arginine-tryptophan peptides affects their uptake and intracellular localization in live mammalian cells, as well as their ability to inhibit bacterial growth. Here, we use quartz crystal microbalance with dissipation monitoring to assess the induced changes caused by binding of the three peptides to supported model membranes composed of POPC, POPC/POPG, POPC/POPG/cholesterol or POPC/lactosyl PE. Our results indicate that the tryptophan position in the peptide sequence affects the way these peptides interact with the different model membranes and that the presence of cholesterol in particular seems to affect the membrane interaction of the peptide with an even distribution of tryptophans in the peptide sequence. These results give mechanistic insight into the function of these peptides and may aid in the design of membrane-active peptides with specified cellular targets and actions.

**Electronic supplementary material:**

The online version of this article (doi:10.1007/s00249-014-0958-9) contains supplementary material, which is available to authorized users.

## Introduction

Cell-penetrating peptides (CPPs) and antimicrobial peptides (AMPs) are two groups of membrane-active peptides (Bechara and Sagan 2013; Fjell et al. 2012; Lohner 2009; Madani et al. 2011). Whereas CPPs have gained extensive interest because of their ability to carry macromolecular cargo across cellular membranes, AMPs have emerged as promising candidates for treatment of antibiotic-resistant bacteria. CPPs and AMPs, however, have many properties in common, and many CPPs can also function as AMPs and vice versa (Henriques et al. 2006; Splith and Neundorf 2011). CPPs and AMPs are short, usually between 10 and 30 amino acids, cationic peptides, often rich in the amino acids arginine and tryptophan (Epand and Vogel 1999; Friedrich et al. 2000; Heitz et al. 2009; Splith and Neundorf 2011; Strömstedt et al. 2010). The function of the peptides, being cell-penetrating or bactericidal, depends on the amino acid sequence and the composition of the target plasma membrane (Bahnsen et al. 2013; Nan et al. 2011; Staubitz et al. 2001; Wang et al. 2010). The strong resemblance between these two peptide classes is being investigated to elucidate the molecular function of these peptides. Delivery of cargo, as well as inhibition of bacterial growth, involves the initial binding of the peptide to the plasma membrane. By studying how peptides with dual function bind to and further interact with membranes, new insights into the mechanism of action may be gained and eventually give guidance for the design of peptides with specific functions and cell membrane specificity.

For the study of peptide-membrane interactions, surface-sensitive analytical techniques using supported model membranes have evolved as interesting complements to the more commonly used bulk techniques applied to liposomes in solution. When studying the interaction between peptides and supported lipid membranes, the real-time kinetics and dynamics of the interaction can be monitored (see, e.g., Cho et al. 2010; Piantavigna et al. 2011). In general, the sensitivity of surface-sensitive techniques with respect to mass uptake is lower compared to fluorescence-based methods, for example, but structural rearrangements of the lipid membrane, such as rupture or fusion of liposomes (Keller and Kasemo 1998; Merz et al. 2008; Richter et al. 2006), alterations in lipid packing and phase transition (Mashaghi et al. 2008), or the formation of protrusions from (Domanov and Kinnunen 2006; Machan et al. 2010) or holes in (Briand et al. 2010; Machan et al. 2010) the membrane, can be readily detected. Thus, by using surface-sensitive techniques, it is possible to detect the changes that a membrane may undergo upon peptide binding, shifting the focus somewhat from the peptide itself to the changes that the binding peptide induces to the membrane. Hence, new, valuable insights into the mechanisms of peptide-membrane interactions can be gained. Investigations of peptide-membrane interactions and, in particular, the effect of specific amino acid sequences using surface-sensitive techniques are however still scarce.

We have previously shown that the number and, even more interestingly, the position of tryptophan in the peptide sequence of a series of arginine-tryptophan peptides can affect both their uptake efficiency into live mammalian cells and their ability to inhibit bacterial growth (Rydberg et al. 2012a, b). The uptake into cells was favored by an even distribution of tryptophans, while the peptides with an accumulation of tryptophans at the N-terminus of the peptide sequence seemed to be more efficient as antibacterial agents. Therefore, we are now investigating whether the biological differences observed can be explained by variations in how these peptides interact with model membranes of different lipid compositions.

**Fig. 1:**
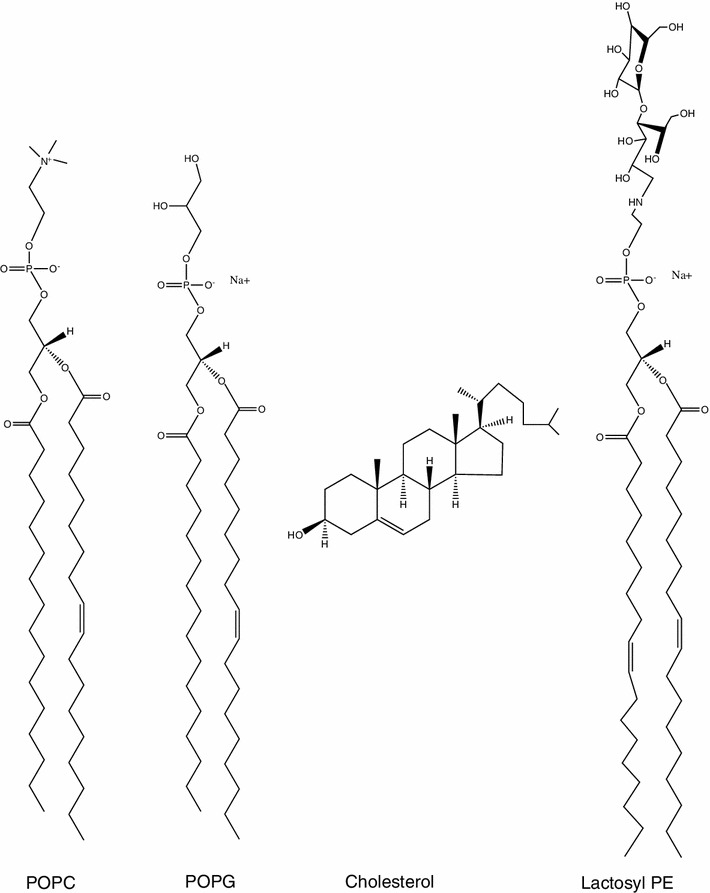
Chemical structure of lipids and glycolipids used in this study

Here, we compare peptide-membrane interactions of three arginine-tryptophan peptides, all containing eight arginines and four tryptophans that are placed either at the N-terminus (WWWWRRRRRRRR), in the middle of the sequence (RRRRWWWWRRRR) or evenly distributed in the sequence (RWRRWRRWRRWR) (Table 1). The interactions between these peptides and neutral POPC membranes as well as negatively charged membranes composed of POPC and either POPG, POPG and cholesterol, or the glycolipid lactosyl PE (Fig. 1), were monitored using the quartz crystal microbalance with dissipation (QCM-D) monitoring technique (Rodahl et al. 1995). We aimed to study how different membrane constituents including cholesterol, present in mammalian but not in bacterial membranes, and membrane-associated carbohydrates, here in the form of the glycolipid lactosyl PE, would affect the peptide-membrane interaction. QCM-D gives information about changes in the mass deposition, seen as shifts in the resonance frequency of the crystal, as well as changes in the viscoelastic properties, seen as shifts in the dissipation of energy during the crystal oscillation. This technique enables the study of peptide-membrane interactions without the need for labeling either the peptide or the membrane, as for example in a previous study of the antibacterial action of the well-known cell-penetrating peptide Tat (Piantavigna et al. 2011). In complementary experiments, we have investigated the distance between peptides binding to a POPC/POPG lipid membrane as well as the ability of the peptides to aggregate liposomes. By studying the interaction of the three different peptides with lipid membranes of different compositions, we provide a physicochemical characterization toward the molecular understanding of the variations observed in cell assays among the three peptides with different tryptophan patterning.

**Table 1:** Peptide sequences, relative uptake efficiency, relative cytotoxicity and relative antibacterial effect

Peptide	Sequence	Uptake efficiency CHO cells^a^	Cytotoxicity CHO cells^b^	Antibacterial effect^c^
W_4_R_8_	WWWWRRRRRRRR	+	+++	+++
RWR	RRRRWWWWRRRR	++	+	++
RWmix	RWRRWRRWRRWR	+++	+	++

^a^Uptake efficiency of 5-FAM labeled peptide in live CHO cells (Chinese Hamster Ovarian cells) measured with flow cytometry (Rydberg et al. 2012b)

^b^Peptide-induced cytotoxicity of CHO cells measured with flow cytometry (Rydberg et al. 2012b)

^c^Peptide-induced growth inhibition of *S. aureus* and *S. pyogenes* (Rydberg et al. 2012a)

## Materials and methods

### Materials

The synthetic lipids (Fig. 1), 1-palmitoyl-2-oleoyl-*sn*-glycero-3-phosphocholine (POPC), 1-palmitoyl-2-oleoyl-*sn*-glycero-3-phospho-(1′-*rac*-glycerol) (POPG), cholesterol and 1,2-dioleoyl-*sn*-glycero-3-phosphoethanolamine-*N*-lactosyl (lactosyl PE) were purchased from Avanti Polar Lipids (Alabaster, AL, USA). For the circular dichroism and fluorescence measurements, POPC and POPG were purchased from Larodan Fine Chemicals (Malmö, Sweden). The RW peptides (>95 % purity), unlabeled and labeled at the N-terminus with 5-carboxyfluorescein (5-FAM), were purchased from Innovagen (Lund, Sweden). The peptides were dissolved in water, and stock solutions of 100 µM were prepared. The peptide concentration was determined with UV–Vis absorbance (using ε_280nm_ of 5,690 M^−1^ cm^−1^ per W or ε_498nm_ of 78,000 M^−1^ cm^−1^ per carboxyfluorescein molecule). Phosphate-buffered saline (PBS) (0.01 M phosphate buffer, 0.0027 M potassium chloride and 0.137 M sodium chloride, pH 7.4) with and without the addition of 10 mM magnesium chloride as well as 10 mM phosphate buffer (Na_2_HPO_4_/NaH_2_PO_4_, pH 7.4) were prepared in water. The buffers were degassed prior to the surface-sensitive experiments. Water was deionized (resistivity >18 MΩ cm) and purified using a MilliQ plus unit (Millipore, France).

### Liposome preparation

POPC, POPG, cholesterol and lactosyl PE were dissolved in chloroform. For QCM-D experiments, lipid films were prepared by mixing solutions of desired lipid composition in round-bottomed flasks. Chloroform was evaporated under a gentle stream of N_2_. To remove any residual solvent, the lipid film was put under vacuum for 2 h. Liposomes were formed by vortexing the lipid film with PBS, followed by extrusion 21 times through polycarbonate membranes (Whatman/GE Healthcare) with 30-nm pore diameter. The average diameter of the liposomes was measured by dynamic light scattering (DLS) using a Malvern Instruments Zetasizer Nano-ZS (Malvern, UK) and was typically around 90-100 nm. For circular dichroism (CD) and fluorescence-quenching experiments, lipids were mixed at the desired composition in round-bottom flasks, and chloroform was evaporated from the lipid solution using a rotary evaporator. After vortexing with 10 mM phosphate buffer, the liposomes were subjected to five cycles of freezing and thawing in N_2_ (l) and 50 °C, respectively. For the liposomes used in the fluorescence-quenching experiments and the CD measurement, polycarbonate membranes with 100-nm pore diameter were used, and the average diameter of these liposomes was measured to be around 140 nm using DLS.

### Quartz crystal microbalance with dissipation monitoring (QCM-D)

QCM-D measurements were performed using a Q-Sense E4 instrument (Q-Sense, Sweden). SiO_2_-coated AT-cut quartz sensors, with a fundamental frequency of 5 MHz, were purchased from Q-Sense AB. The sensors were cleaned in 10 mM sodium dodecyl sulfate aqueous solution over night. Just prior to the experiment, the sensors were rinsed thoroughly with water, dried under N_2_ and treated with UV/ozone for 30 min. The measurements were carried out at 22 °C. Frequency and dissipation shifts (Δ*f* and Δ*D*) were recorded at the 3rd, 5th, 7th, 9th and 11th harmonics. As very similar frequency and dissipation data were obtained at the different harmonics, which is characteristic for rigid layers in QCM-D, only the results obtained at the seventh harmonic are presented in the graphs. Frequency shifts were normalized to the fundamental frequency by dividing with the overtone number. The mass deposition of peptide on the lipid surface was estimated using the Sauerbrey equation (Eq. 1) (Sauerbrey 1959)

1 $$ \Delta m = - C_{\text{QCM}} \frac{{\Delta f_{n} }}{n} $$

where Δ*m* is the adsorbed mass on the surface, *C*_QCM_ is the mass sensitivity constant (17.7 ng cm^−2^ Hz^−1^, for the 5 MHz quartz sensors used in our study), and Δ*f*_n_ is the change in the resonance frequency at the *n*th harmonic. The Sauerbrey equation is valid for rigid films and was used to estimate the mass of the membrane systems, which were characterized by low dissipation shifts.

### Investigation of peptide-membrane interaction using QCM-D

Supported lipid membranes were formed on SiO_2_-coated sensors. The frequency shift, Δ*f*, was typically between −25 and −27 Hz, and the dissipation shift, Δ*D*, was <0.5 × 10^−6^, indicating the formation of good quality membranes (Keller and Kasemo 1998). In short, solutions were added to the crystal in the following order: PBS, PBS-MgCl_2_, liposomes freshly diluted in PBS-MgCl_2_, PBS-MgCl_2_ and PBS. MgCl_2_ was added to facilitate the formation of negatively charged membranes (Kunze et al. 2009). For POPC membranes, the liposomes were diluted in PBS without further addition of MgCl_2_. The flow rate was 100 µl/min. After membrane formation, peptide solution diluted in PBS (5 µM) was added for 20 min at a rate of 50 µl/min, after which the flow was stopped for 60 min for measuring for a longer period while saving the peptide material. Measurements in which the peptide was added for 40 min instead of 20 min were also performed, as well as measurements in which the peptide was directly added to the SiO_2_ surface of the QCM-D sensor. The measurements were repeated three to four times, using the same stock solutions of peptides and lipids, but performed on different days, using different QCM-D equipment and different solutions and suspensions.

### Fluorescence self-quenching of the RW peptides binding to liposomes

To verify the QCM-D results and to approximate the maximal binding of the peptides to the lipid surface, fluorescence intensity and self-quenching of the carboxyfluorescein-labeled peptide were monitored upon binding to liposomes. A 1.25-µM solution of fluorescently labeled peptide was titrated with 2-µl steps of a 10 mM solution of POPC/POPG 80/20 mol % liposomes. Peptides and liposomes were both diluted in a 10-mM phosphate buffer, pH 7.4. The fluorescence intensity was measured using a Cary Eclipse Fluorescence Spectrophotometer (Varian). The excitation was 480 nm and emission 490–700 nm. From the experimental values, the amount of peptide bound per cm^2^ of lipid surface was calculated (for calculation, see Supplementary Information).

### Measurement of peptide-induced aggregation of liposomes using dynamic light scattering (DLS)

To assess peptide-induced aggregation, POPC/POPG liposomes were titrated with increasing amounts of peptide, and the aggregate size was measured using DLS. The liposomes were diluted in PBS to a concentration of 46 µM, and the peptide solution, 100 µM dissolved in water, was then added in steps of 5 µl (or 10 µl for RWmix) until all liposomes had aggregated. Aggregation was seen by a shift in the size from around 90 nm for well-dispersed liposomes to around 1,000 nm for bigger aggregates (a graphic illustration of these shifts is presented in Fig. S1, Supplementary Information). For the larger aggregates, the sizes are rough estimations of the actual sizes because of the polydispersity of the sample. After each peptide addition, three DLS measurements were performed, each of ten runs.

### Assessment of peptide secondary structure using circular dichroism (CD)

Circular dichroism was used to study the secondary structure of the peptides when bound to liposomes of POPC/POPG and POPC/POPG/cholesterol, respectively. To investigate any occurrence of slow time dependence or lag phase in the binding event, CD was also measured on the POPC/POPG liposomes at 80 min after peptide addition. Peptides and liposomes were diluted in a 10-mM phosphate buffer. Spectra were recorded between 185 and 270 nm on a Chirascan Circular Dichroism Spectrometer (Applied Photophysics, UK). Time per point was 0.500 s. The samples were measured at 20 °C in a 2-mm pathlength quartz cell. For each sample, 20 scans were recorded and averaged. Spectra were corrected for background contributions by subtraction of appropriate blanks. The peptide concentration was 5 μM, and the peptide-to-lipid molar ratio was 1:100. The peptide secondary structure was evaluated by comparison with standard reference spectra (Kelly et al. 2005; Nordén et al. 2010).

## Results

### Interaction of the RW peptides with supported lipid membranes of different compositions

The surface-sensitive technique QCM-D was used as a tool to investigate the membrane interaction of the three RW peptides with supported lipid membranes of varying composition. We aimed to investigate the importance of electrostatics for efficient peptide binding by comparing neutral and negatively charged membranes. We also wanted to investigate how cholesterol, which is found in mammalian membranes but not in bacterial membranes, might affect the peptide-membrane interaction. Finally, we were intrigued to investigate the interaction between the peptides and membranes containing glycolipids, since surface carbohydrates in general are important for the interaction between membrane-active peptides and mammalian and bacterial membranes, and since the expression of glycolipids, in particular, has been associated with cancer progression. We wanted to use a commercially available glycolipid, and we also wanted this glycolipid to show similarity with POPG as well as being negatively charged. We therefore chose the glycolipid lactosyl PE, although this particular glycolipid originates from plants. In QCM-D, a mass deposition of peptide on the lipid surface is seen as a decrease in frequency, and changes in the viscoelastic properties of the lipid membrane upon peptide binding are related to changes in the dissipation signal. The peptide concentration of 5 µM, used for the surface interaction experiments, was chosen since the peptides at this concentration show evident biological responses in regard to both cellular uptake and bacterial growth inhibition, as seen in our previous results (Rydberg et al. 2012a, b).

**Fig. 2:**
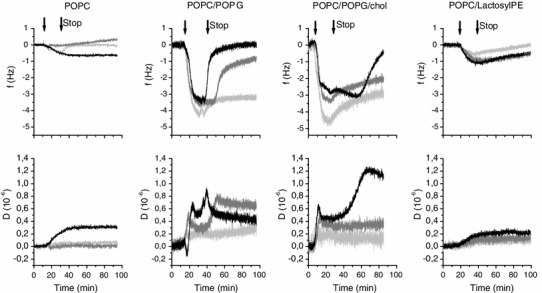
QCM-D frequency, Δ*f*, and dissipation, Δ*D*, shifts as a function of time for the RW peptides when binding to four different lipid membranes. Typical graphs are shown. The *first arrow* from the *left* (*t* = 20 min) indicates addition of peptide to the membrane, and the *second arrow* (*t* = 35 min) indicates where the flow is stopped. The peptide concentration was 5 µM. W_4_R_8_ is presented in *light gray*, RWR in *gray* and RWmix *dark gray*. The lipid compositions were POPC, POPC/POPG 80/20 mol %, POPC/POPG/cholesterol 75/20/5 mol % and POPC/lactosyl PE 80/20 mol %

**Fig. 3:**
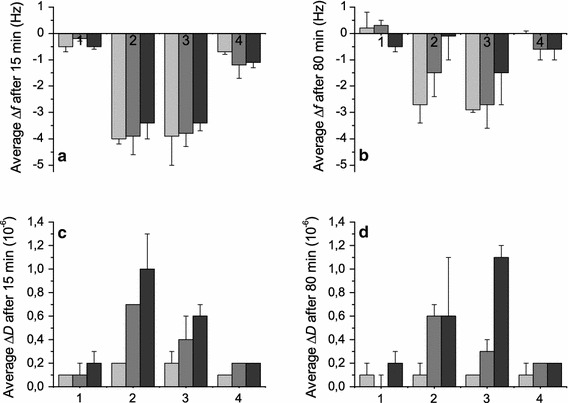
Average QCM-D frequency and dissipation shifts (Δ*f* and Δ*D*) at different time points (15 and 80 min) after the addition of peptide to the membrane (see also Table S1 in Supplementary Information). The *top left graph* (**a**) shows Δ*f* after 15 min, the *top right graph* (**b**) shows Δ*f* after 80 min, the *lower left graph* (**c**) shows Δ*D* after 15 min, and the *lower right graph* (**d**) shows Δ*D* after 80 min. The 15-min time point corresponds to the maximal frequency shift reached upon peptide binding. W_4_R_8_ is presented in *light gray*, RWR in *gray* and RWmix in *dark gray*. The four different lipid membranes are labeled as follows: (1) POPC, (2) POPC/POPG 80/20 mol %, (3) POPC/POPG/cholesterol 75/20/5 mol % and (4) POPC/lactosyl PE 80/20 mol %. Values are averages of 3–4 measurements. *Error bars* represent standard deviations

Starting with the neutral POPC membrane (Figs. 2, 3), the mass deposition for all three peptides was very low as seen by low frequency shifts of around or less than −0.5 Hz. The corresponding shifts in dissipation were also low, around or less than 0.2 × 10^−6^ (Fig. 2 and Table S1 in Supplementary Information), suggesting weak membrane interaction. It should be pointed out that low-molecular-weight peptides, such as the RW peptides, generally result in small signals in QCM-D, unless major rearrangement of the model membrane occurs. At 80 min after peptide addition, the frequency signals are close to the original (before peptide addition). The poor binding of the cationic peptides to the POPC membrane demonstrates that electrostatic interaction is required for efficient membrane binding of these peptides.

When negatively charged lipids, 20 mol % of POPG, were included in the membrane (Figs. 2, 3), significant responses in both Δ*f* and Δ*D* were observed upon peptide addition. The Δ*f* and Δ*D* signals for the different harmonics were virtually the same; therefore, only the results from the seventh harmonic are presented. The frequency shifts were of similar shape and magnitude for the three peptides, with the exception of W_4_R_8_ showing a slightly higher mass deposition. The shifts in frequency around 15 min after peptide addition were around 3.5–4 Hz, which corresponds to a mass deposition of around 30–35 ng/cm^2^ [using the Sauerbrey equation (Eq. 1) and the assumption that 50 % of the mass deposition is water (Edvardsson et al. 2009)]. When the flow was stopped, approximately 20 min after peptide addition, the frequency responses unexpectedly decreased, or, as for RWmix, even returned to values close to the original, resulting in U-shaped frequency curves.

Since the frequencies of RWmix binding to the POPC/POPG membranes return to values close to the original upon stopping the flow, which would imply loss of mass, it was investigated whether peptide remained bound to the membrane surface. To do so, POPC/POPG liposomes were added to the membranes that had been exposed to peptide for 60 min, followed by a buffer rinse. We observed that the liposomes were bound to the membranes that had been exposed to all three peptides, whereas liposomes did not bind to unexposed membranes (Supplementary Information). The amount of binding liposomes varied between the peptides, possibly indicating variations in the amount of peptide at the surface or reflecting the ability of the peptides to aggregate liposomes at the surface. The results clearly indicate that peptide is still present at the membrane surface after the flow stop, also in the cases where the QCM-D signals suggest mass removal upon stopping the flow. Additionally, these results point to a possible flow dependency in the peptide-membrane interaction, which is addressed further below.

Whereas the frequency shifts are similar among the three peptides interacting with the POPC/POPG membrane, the shifts in dissipation clearly differ, as seen in Fig. 2, regarding both amplitude and shape, with W_4_R_8_ showing low dissipation, whereas RWR, and especially RWmix, shows interesting patterns of peaks and minima, possibly indicating structural rearrangements in the membrane. Figure 3 shows that the average dissipation values are the highest for RWmix at around 1 × 10^−6^, followed by RWR, and the lowest for W_4_R_8_ at around 0.2 × 10^−6^. After the flow has been stopped, the dissipation shifts stabilize.

Since mammalian cell membranes contain cholesterol, whereas bacterial membranes do not, we added 5 mol % of cholesterol to the POPC/POPG membrane to investigate how it would affect the peptide-membrane interaction of the three RW peptides. With the addition of cholesterol to the membrane, W_4_R_8_ shows a slight increase in membrane deposition compared with the other two peptides, seen as a slightly higher shift in frequency. The amount of peptide that binds to the surface otherwise seems to be similar comparing the POPC/POPG and POPC/POPG/cholesterol membranes (Figs. 2, 3). The shapes of the frequency curves of W_4_R_8_ and RWR are very similar. The frequency signal of RWmix markedly differs compared to both the other two peptides and the non-cholesterol containing membrane, with a slight increase in frequency after the flow has been stopped, followed by the frequency decreasing toward the initial values after around 60 min. The dissipation follows the same trend. The dissipation shifts for W_4_R_8_ and RWR are similar to that of W_4_R_8_ of POPC/POPG. For RWmix, there is a peak in dissipation when peptide is added to the membrane, followed by a small decrease. This particular behavior of RWmix, compared with the other two peptides, is clearly visualized when the dissipation is plotted as a function of frequency (Fig. S2, Supplementary Information). After the flow is stopped, interestingly, the dissipation increases conspicuously to values above 1 × 10^−6^, indicating rearrangements in the membrane.

Since the surfaces of mammalian and bacterial cells are covered with carbohydrates, we were also intrigued about adding a carbohydrate to the membrane. We chose the commercially available lactosyl PE (Fig. 1), a doubly saturated lipid with a negatively charged head group where a lactose unit is attached, to investigate whether also small non-ionic sugars may have an impact on the binding of the RW peptides to membranes. Even though lactosyl PE, to the authors’ knowledge, has not been used before in the formation of supported lipid membranes, the preparation of glycolipid-containing supported membranes by liposome fusion has been previously described (Kunze et al. 2013; Rydell et al. 2009), and similarly, lactosyl PE-containing supported membranes were readily formed. As seen in Figs. 2 and 3, the shifts in frequency for the peptides binding to the POPC membrane containing 20 mol % of the glycolipid are rather low, with values around −1 Hz, which decrease with time, indicating low peptide binding. Furthermore, the dissipation is small, with only minor shifts upon peptide addition, however stable with time, also pointing to weak peptide-membrane interactions.

### Investigation of the role of the flow conditions used in the QCM-D measurements

**Fig. 4:**
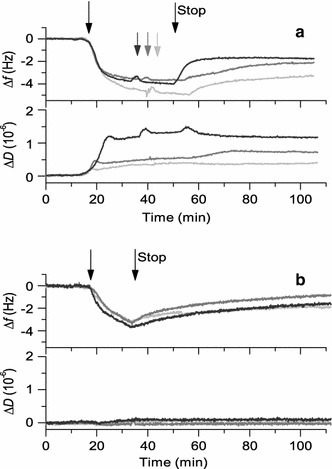
Illustration of the importance of the experimental conditions for the QCM-D results. In the *upper graph* (**a**), frequency and dissipation shifts are presented for the binding of the RW peptides to POPC/POPG membranes, with the time during which the peptide solution is added before stopping the flow being doubled compared to the experiment in Fig. 2. In the *lower graph* (**b**), frequency and dissipation shifts obtained for RW peptide binding directly to a SiO_2_ surface are shown. W_4_R_8_ is presented in *light gray*, RWR in *gray* and RWmix in *dark gray*. The *arrows* to the very *left* (*t* = 20 min) indicate addition of peptide to the membranes, and the *arrows* labeled “Stop” indicate where the flow was stopped. *Gray arrows* in **a** indicate short (1 min) stops in flow to refill the peptide solution. For both **a** and **b**, it is seen that the frequency shift decreases when the flow is stopped

To further investigate the QCM-D frequency shifts observed 20 min after peptide was added to the lipid membranes and as the flow was stopped, the peptide-membrane interaction experiments were repeated under a different flow condition. This time, the flow of peptide solution was prolonged from around 20 min to around 40 min. Since the dependence was seen in particular for the POPC/POPG membrane, this lipid composition was used. As seen in Fig. 4a, the frequency signals decrease when the flow is stopped also after 40 min of peptide exposure. The decrease is however not as large as in the experiment described above where the flow was stopped after 20 min.

Furthermore, peptide was also added directly to the silica-coated QCM-D sensor without any lipid membrane. Figure 4b shows that when the flow is stopped after approximately 20 min of peptide flow, the frequency signals decrease toward reaching a plateau at Δ*f* of around 1–2 Hz. In this case, with no lipid membrane present, there is no difference in the frequency signal among the three peptides. Also, there is no shift in dissipation upon peptide binding, indicating that the peptides obtain a flat configuration relative to the surface (Rodahl et al. 1995).

Taken together, these results support the assumption that the decrease in frequency seen is due to the flow stop and to structural rearrangements in the membranes. In particular, these results verify that the intrinsic properties of the peptides influence their interaction with the membranes as well as the effect of the flow stop on this interaction, as all peptides yielded similar results on the silica surface.

### Assessment of peptide secondary structure upon binding to liposomes

**Fig. 5:**
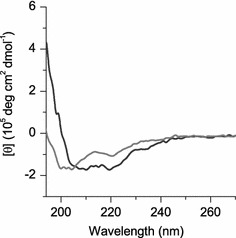
CD measurement of secondary structures of RWmix (*light grey*) and its twice as long analog RWmix2 (*dark grey*), showing that the α-helical signal is higher and more pronounced for the longer peptide

To investigate whether the differences seen in frequency and dissipation signals for membranes with and without cholesterol are caused by changes in peptide secondary structure, CD was measured for the three peptides binding to POPC/POPG liposomes with and without the addition of 5 mol % cholesterol (Supplementary Fig. S3). Only minor variations in the peptide secondary structure were noticed, indicating that the different results observed for membranes with and without cholesterol in the QCM-D measurements (Fig. 2) are caused by alterations in the membrane properties rather than changes in the peptide secondary structure. As described previously, RWR and W_4_R_8_ adopt random coil structures upon membrane binding, whereas RWmix adopts a more pronounced secondary structure with helix-like features. We hypothesized that the RWmix does form an α-helix, but that the limited length of the peptide causes the low CD signal. Therefore, CD was also measured for a peptide with a double RWmix sequence (RWRRWRRWRRWRRWRRWRRWRRWR). Figure 5 shows that this peptide (RWmix2) indeed adopts an evident α-helix when interacting with POPC/POPG liposomes.

### Assessment of maximal binding of RW peptides to liposomes using fluorescence self-quenching

**Fig. 6:**
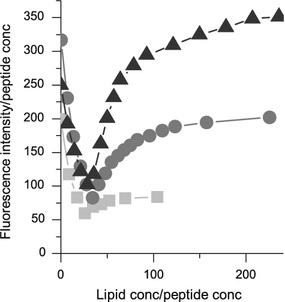
Fluorescence intensity of 5-FAM labeled peptides binding to POPC/POPG liposomes as a function of the peptide-to-lipid ratio. W_4_R_8_ is presented as *light gray squares* (*filled square*), RWR as *gray circles* (*filled circle*) and RWmix as *dark gray triangles* (*filled triangle*). Fluorescence minima, where fluorophore self-quenching is the highest and the distance between the peptides is the smallest, are found at peptide to lipid ratios of about 1:30 for all three peptides

The maximal peptide binding to POPC/POPG membranes was further evaluated using fluorescence self-quenching experiments, where increasing amounts of POPC/POPG liposomes were added to carboxyfluorescein-labeled RW peptides in solution. The peptide-to-lipid ratio for the RW peptides, for which self-quenching of the carboxyfluorescein fluorophores was the highest, i.e., the fluorescence intensity was the lowest, were around 1:30 for all three peptides (Fig. 6). Fluorescein is believed to self-quench as a result of two processes: first, the formation of non-fluorescent fluorescein dimers; second, energy transfer from fluorescein monomers to the non-fluorescent dimers when the distance between them is equal or less than the critical Förster distance, which for FITC fluorophores is about 55 Å (Chen and Knutson 1988; Runnels and Scarlata 1995). When liposomes (POPC/POPG) are added stepwise to the fluorescently labeled peptide in solution, the fluorescence intensity first decreases and then, at peptide-to-lipid ratios of about 1:30, increases again. This indicates that when liposomes are added at ratios below and up to 1:30, the peptides bind at such a high concentration to the membrane surface that fluorophores self-quench. Above 1:30, the membrane surface concentration of peptide decreases, leading to larger distances between the binding peptides and decreased self-quenching. It is also possible that at this peptide-to-lipid ratio, the peptide binding is sufficiently high to cause charge neutrality. This would mean that the maximum binding capacity of the peptide to the membrane is reached, which in turn may cause aggregation of the liposomes. For the concentrations where fluorescence minima were noticed, where the maximum amount of peptide is expected to be bound to the lipid membrane, the amount of peptide bound per cm^2^ (surface concentration) was calculated (see Supplementary Information). For W_4_R_8_, the number of peptide molecules per cm^2^ was 10.0 × 10^12^, for RWR it was 7.9 × 10^12^, and for RWmix it was 9.5 × 10^12^, or in mass per cm^2^, 35 ng/cm^2^ for W_4_R_8_, 26 ng/cm^2^ for RWR and 32 ng/cm^2^ for RWmix. These results correspond well to the mass deposition estimated from the QCM-D experiments, indicating that for the peptide concentration tested, near binding saturation is reached. At lipid concentrations above maximal binding, the degree of quenching interestingly differs among the three peptides, possibly as a result of liposome aggregation.

### Peptide-induced aggregation of POPC/POPG liposomes

**Fig. 7:**
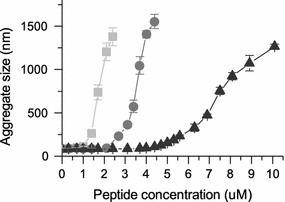
The approximate hydrodynamic diameters (estimated from DLS) of POPC/POPG liposome aggregates as a function of increasing peptide concentration. The peptides cause aggregation to different extents, with most aggregation seen for W_4_R_8_ (*light gray* *filled square*), followed by RWR (*gray* *filled circle*) and then RWmix (*dark gray* *filled triangles*). The initial lipid concentration was 46 µM.* Error bars* represent standard deviations of three measurements

Liposome aggregation was studied to verify whether the differences seen between the peptides in reaching plateau levels in the fluorescence quenching experiments were due to variations in the way the peptides induced aggregation of the liposomes. To assess aggregation, the particle size of POPC/POPG liposomes was measured using DLS after the addition of increasing amounts of peptide. As seen in Fig. 7, all three RW peptides induce aggregation of liposomes, but to different extents. The peptide that caused the most aggregation, at the lowest peptide concentration, was W_4_R_8_, followed by RWR. A substantially larger amount of RWmix was needed for the same degree of aggregation to occur as compared with W_4_R_8_. The peptide-to-lipid ratio for the last data point, where all liposomes had aggregated, was around 1:20 for W_4_R_8_, 1:10 for RWR and 1:5 for RWmix. Since DLS results for polydisperse samples are difficult to evaluate, the sizes of the complexes are only very approximate.

## Discussion

The three RW peptides, W_4_R_8_, RWR and RWmix, all contain four tryptophans and eight arginines. Even though they are built up of the same 12 amino acids, their biological action differs, especially regarding their cell-penetrating properties in mammalian cells (Table 1). It might be justified to assume that the biological differences seen derive from alterations in how these peptides interact with membranes, and we therefore wanted to investigate their interaction with membranes of different compositions using the surface-sensitive QCM-D technique. QCM-D not only allows for the detection of adsorbed mass, but also for characterization of the viscoelastic properties of molecular layers on the sensor surface.

### Binding of the RW peptides to membranes of different composition

The QCM-D data give us information about how the position of the amino acids and altered lipid composition affect the peptide-membrane interaction. For the POPC/POPG membrane, variations in how the three RW peptides interact with lipid membranes were monitored (Fig. 2). Evident differences in the frequency signals are seen between the peptides, and W_4_R_8_ shows a slightly higher mass deposition and lower dissipation shifts compared to RWR and RWmix.

With the addition of cholesterol to the membrane, the signals of RWmix clearly differ from those of RWR and W_4_R_8_, regarding both frequency and dissipation. RWmix is the only one of the peptides that forms an α-helix when interacting with the lipid membranes. It is therefore reasonable to believe that all four tryptophans of this peptide are positioned in the membrane, which might not be the case for the other two peptides. This points to the possibility that there might be a specific interaction between the tryptophans and the cholesterol moieties in the membrane. The potential existence of such an interaction has been debated. Raghuraman and Chattopadhyay (2004), for example, have provided evidence that the cholesterol analog dehydroergosterol is preferentially localized close to the tryptophan moiety of melittin. By contrast, Holt et al. (2008) have found that there is no preferential interaction between cholesterol and the tryptophans of the WALP23 peptide. Although there is no consensus yet regarding the specific interaction between tryptophan and cholesterol, cholesterol still undeniably affects the properties of lipid membranes by altering membrane thickness and internal fluidity, generally making the membrane more rigid. In addition, the lack of cholesterol in bacterial membranes contributes to specificity between bacterial and mammalian membranes. The evidently higher uptake of RWmix in live mammalian cells and the higher degree of endocytotic uptake (Rydberg et al. 2012b) compared to RWR and W_4_R_8_ might thus possibly be related to the helical structure of the peptide and the cholesterol content of the membrane.

For the POPC and POPC/lactosyl PE membranes, low mass depositions and dissipation shifts are seen. The smallest QCM-D frequency shifts that can be measured with good resolution from the noise level are about 0.5 Hz using the present equipment, meaning that the sensitivity of QCM-D is in the range of 1 ng/cm^2^. For POPC and POPC/lactosyl PE, the measured values are at the border of, or just above, this resolution, emphasizing the importance of assistance from electrostatic attraction for efficient binding. In addition, we cannot rule out the possibility of defects in the membrane that may potentially influence the results when the binding is weak. Lactosyl PE, the doubly saturated lipid carrying a negatively charged head group where a lactose is attached, was included in the POPC membrane to investigate how small uncharged sugars may affect the binding of the RW peptides to membranes. Extracellular carbohydrates are suggested to be important for the mechanism of action of membrane-active peptides. For example, proteoglycans have been associated with the uptake of CPPs into cells (Heitz et al. 2009; Åmand et al. 2012), and the lipopolysaccharides of the outer membrane of gram-negative bacteria have been associated with the antibacterial effect of AMPs (Domingues et al. 2009; Epand and Vogel 1999; Yeaman and Yount 2003). Binding of the peptides to carbohydrates may also impair the antibacterial effect by hindering the peptides from reaching the bacterial membrane. A similar function may be seen for the membrane with lactosyl PE, where the two sugar rings of lactose seem to sterically hinder efficient interaction between the positively charged peptides and the negatively charged head group of the glycolipid. It has also been reported that there may be a specific interaction between tryptophans and glycosaminoglycans (Bechara et al. 2013). However, considering the lower mass deposition on the lactosyl PE-containing membrane, it also appears feasible that the interaction between tryptophans and short, uncharged glucose chains should not be strong enough for efficient binding.

### The QCM-D results depend on the flow conditions and the intrinsic properties of peptides and membranes

The decrease in frequency seen in the QCM-D results for all three peptides around 20 min after peptide addition and upon stopping of the flow, for both the POPC/POPG and POPC/POPG/cholesterol membranes (Fig. 2), is dependent on the altered flow conditions. The reproducibility of these results, together with the experiment with twice as long flow of peptide solution before the flow stop and the examination of peptide bound directly to the SiO_2_ surface of the sensor, strongly suggests a flow dependence of the peptide-membrane interaction, although this is very unusual and unexpected. As the interaction between the peptides and the lipid membranes seemingly had reached equilibrium, one would expect the signals to stay constant as the flow is stopped. One possible explanation for this finding is that there is a flow dependency for the interaction between the peptide and the membrane. It might also be possible that the process is not at equilibrium despite the constant signal achieved after around 15 min. In addition, we cannot rule out non-ideal mixing conditions in the measurement cell, leading to a decrease in peptide concentration over the center of the sensor surface upon stopping the flow. Flow dependencies of QCM-D signals have rarely been reported other than as a means to improve on mass transport limitations. It can however be noted that alterations of the shear flow may cause a rupture of liposomes (Jönsson et al. 2009) or accumulation of membrane constituents at the rear edge of the membrane (Jönsson et al. 2011). The flow stop may thus result in decreased shear forces, in turn leading to structural rearrangements at the membrane surface. The flow stop might in this case also lead to slower diffusion of material to and away from the membrane, compared to under flow conditions, thus revealing processes that still occur in the membrane despite the apparent equilibrium condition.

Interestingly, this flow stop phenomenon seems to be of different importance for different peptides and membranes. Considering the POPC/POPG and POPC/POPG/cholesterol membranes, the stop flow effect is more pronounced in the absence of cholesterol. The presence of cholesterol in the lipid membrane instead appears to result in a more stable interaction, where only small changes in the QCM-D responses were observed upon stopping the flow around 20 min after peptide addition. This might be a result of the higher rigidity of the membrane caused by the cholesterol molecules, an effect known from various contexts (Boggs and Hsia 1972; Niemelä et al. 2007; Róg et al. 2009). Considering the differences between the peptides, the membrane interaction of RWmix seems to be more affected by the flow stop than those of the other two peptides, suggesting differences in the intrinsic properties of the peptides.

The decrease in mass deposition seen when the flow is stopped could at a first glance be due to the peptide leaving the membrane, as the diffusion conditions are altered upon stop flow. However, the attachment of POPC/POPG liposomes to the supported membrane after peptide addition and flow stop showed that peptide was indeed still present on the surface. Other mechanisms thus seem to be involved as well, and it is therefore plausible to envisage that small amounts of lipid material may be leaving the membrane. It is for example possible that binding of the RW peptides, and especially RWmix, which shows a more pronounced secondary structure and more membrane rearrangement compared with the two other peptides, may cause membrane thinning whereby lipids leave the membrane (Cho et al. 2007). The peptides could possibly also cause a loss of lipid material by detergent-like mechanisms (Strömstedt et al. 2010). In addition, the increase in dissipation suggests that the peptide might also cause protrusions or other structural rearrangements of the membrane (Domanov and Kinnunen 2006), which might lead to the loss of lipids from the surface when the flow is stopped. It is also possible that the peptide binding results in compaction of the membrane, which may cause a loss of water, seen as decreased mass deposition. The lesser extent of the frequency decrease seen when cholesterol is added to the membrane could be explained by the membrane becoming more rigid and less prone to structural rearrangements. The flow stop thus might be used as a tool to investigate the intrinsic properties of peptides and their interactions with membranes.

### The interaction of the RW peptides with POPC/POPG liposomes

In the fluorescence quenching experiments that were performed to verify the mass deposition of the three peptides in the QCM-D experiments, it was seen that the three peptides behaved differently. At lipid concentrations above the maximal binding, the peptides showed different levels of fluorescence quenching, which we hypothesized might be related to their ability to aggregate the liposomes. The aggregation studies support this hypothesis. Aggregation of POPC/POPG liposomes by the addition of the three RW peptides was not unexpected and is often observed for CPPs and AMPs (Domingues et al. 2009; Persson et al. 2001; Rodrigues et al. 2012). However, the distinct variation between the peptides, with W_4_R_8_ causing aggregation already at a very low concentration, whereas a higher concentration of RWR and much higher concentrations of RWmix are needed for the same degree of aggregation, was not expected. Thus, not only the charge neutralization introduced by the +8 charged peptides as described by others (Marquette et al. 2010) (which is the same for all three peptides), but also the position of the tryptophans in the peptide sequence affects the aggregation. W_4_R_8_ is the most amphipathic of the three peptides, with the four tryptophans placed at the N-terminus. The degree of aggregation hence seems to be correlated to the amphipathicity of the peptide: the more amphipathic, the more aggregation. Changing the position of the tryptophans in the RW peptides of course also alters the position of the arginines. Alteration of the position of the tryptophans and arginines may alter both the intramolecular and intermolecular interactions. For example, variations in the amino acid position alter the charge distribution, the ability to form π-cation interactions and the ability to form h-bonds. This can affect the peptide-membrane interaction, and the three peptides may for example have different abilities to cluster membrane lipids (Oreopoulos et al. 2010; Wadhwani et al. 2011). Details of membrane interactions of tryptophan arginine peptides have been described by others (see, e.g., Aliste et al. 2003; Dathe et al. 2004; Nguyen et al. 2011; Shaw et al. 2008). In addition, the varying positions of tryptophans and arginine might influence the ability of the peptides to form complexes, which might affect the interaction with both liposomes in suspension and with supported lipid membranes. Conclusions regarding the involvement of these interactions in the peptide-membrane interaction can however not be drawn from the results presented in this report.

## Conclusions

In conclusion, by use of QCM-D and spectroscopic assays, we here show that the position of tryptophan in the amino acid sequence of three tryptophan-arginine peptides alters the way these peptides interact with lipid membranes of different compositions. We see that in the presence of cholesterol, an even tryptophan distribution results in increased membrane disturbance, which might be related to the evidently higher uptake of this peptide in mammalian cells. We also see that increased separation between the tryptophans and the arginines in the peptide backbone increases the ability to aggregate liposomes. In addition, we suggest that alteration of the flow conditions in QCM-D experiments might be used to distinguish differences in the intrinsic properties of peptides interacting with lipid membranes.

## Electronic supplementary material

Below is the link to the electronic supplementary material.

Supplementary material 1 (DOCX 280 kb)

## References

[CR1] Aliste MP, MacCallum JL, Tieleman DP (2003). Molecular dynamics simulations of pentapeptides at interfaces: salt bridge and cation-pi interactions. Biochemistry.

[CR2] Åmand HL, Rydberg HA, Fornander LH, Lincoln P, Nordén B, Esbjörner EK (2012). Cell surface binding and uptake of arginine- and lysine-rich penetratin peptides in absence and presence of proteoglycans. Biochimica Et Biophysica Acta-Biomembranes.

[CR3] Bahnsen JS, Franzyk H, Sandberg-Schaal A, Nielsen HM (2013). Antimicrobial and cell-penetrating properties of penetratin analogs: effect of sequence and secondary structure. Biochimica Et Biophysica Acta-Biomembranes.

[CR4] Bechara C, Sagan S (2013). Cell-penetrating peptides: 20 years later, where do we stand?. FEBS Lett.

[CR5] Bechara C, Pallerla M, Zaltsman Y, Burlina F, Alves ID, Lequin O, Sagan S (2013). Tryptophan within basic peptide sequences triggers glycosaminoglycan-dependent endocytosis. Faseb J.

[CR6] Boggs JM, Hsia JC (1972). Effect of cholesterol and water on rigidity and order of phosphatidylcholine bilayers. Biochimica et Biophysica Acta.

[CR7] Briand E, Zach M, Svedhem S, Kasemo B, Petronis S (2010). Combined QCM-D and EIS study of supported lipid bilayer formation and interaction with pore-forming peptides. Analyst.

[CR8] Chen RF, Knutson JR (1988). Mechanism of fluorescence concentration quenching of carboxyfluorescein in liposomes—energy-transfer to nonfluorescent dimers. Anal Biochem.

[CR9] Cho NJ, Cho SJ, Hardesty JO, Glenn JS, Frank CW (2007). Creation of lipid partitions by deposition of amphipathic viral peptides. Langmuir.

[CR10] Cho NJ, Frank CW, Kasemo B, Höök F (2010). Quartz crystal microbalance with dissipation monitoring of supported lipid bilayers on various substrates. Nat Protoc.

[CR11] Dathe M, Nikolenko H, Klose J, Bienert M (2004). Cyclization increases the antimicrobial activity and selectivity of arginine- and tryptophan-containing hexapeptides. Biochemistry.

[CR12] Domanov YA, Kinnunen PKJ (2006). Antimicrobial peptides temporins B and L induce formation of tubular lipid protrusions from supported phospholipid bilayers. Biophys J.

[CR13] Domingues MM, Castanho MARB, Santos NC (2009). rBPI(21) Promotes Lipopolysaccharide Aggregation and Exerts Its Antimicrobial Effects by (Hemi)fusion of PG-Containing Membranes. PLoS ONE.

[CR14] Edvardsson M, Svedhem S, Wang G, Richter R, Rodahl M, Kasemo B (2009). QCM-D and reflectometry instrument: applications to supported lipid structures and their biomolecular interactions. Anal Chem.

[CR15] Epand RM, Vogel HJ (1999). Diversity of antimicrobial peptides and their mechanisms of action. Biochimica Et Biophysica Acta-Biomembranes.

[CR16] Fjell CD, Hiss JA, Hancock REW, Schneider G (2012). Designing antimicrobial peptides: form follows function. Nat Rev Drug Discov.

[CR17] Friedrich CL, Moyles D, Beveridge TJ, Hancock REW (2000). Antibacterial action of structurally diverse cationic peptides on gram-positive bacteria. Antimicrob Agents Chemother.

[CR18] Heitz F, Morris MC, Divita G (2009). Twenty years of cell-penetrating peptides: from molecular mechanisms to therapeutics. Br J Pharmacol.

[CR19] Henriques ST, Melo MN, Castanho MARB (2006). Cell-penetrating peptides and antimicrobial peptides: how different are they?. Biochem J.

[CR20] Holt A, de Almeida RFM, Nyholm TKM, Loura LMS, Daily AE, Staffhorst R, Rijkers DTS, Koeppe RE, Prieto M, Killian JA (2008). Is there a preferential interaction between cholesterol and tryptophan residues in membrane proteins?. Biochemistry.

[CR21] Jönsson P, Beech JP, Tegenfeldt JO, Höök F (2009). Mechanical behavior of a supported lipid bilayer under external shear forces. Langmuir.

[CR22] Jönsson P, Gunnarsson A, Höök F (2011). Accumulation and separation of membrane-bound proteins using hydrodynamic forces. Anal Chem.

[CR23] Keller CA, Kasemo B (1998). Surface specific kinetics of lipid vesicle adsorption measured with a quartz crystal microbalance. Biophys J.

[CR24] Kelly SM, Jess TJ, Price NC (2005). How to study proteins by circular dichroism. Biochimica Et Biophysica Acta-Proteins and Proteomics.

[CR25] Kunze A, Svedhem S, Kasemo B (2009). Lipid transfer between charged supported lipid bilayers and oppositely charged vesicles. Langmuir.

[CR26] Kunze A, Bally M, Höök F, Larson G (2013). Equilibrium-fluctuation-analysis of single liposome binding events reveals how cholesterol and Ca2+ modulate glycosphingolipid trans-interactions. Sci Rep.

[CR27] Lohner K (2009). New strategies for novel antibiotics: peptides targeting bacterial cell membranes. Gen Physiol Biophys.

[CR28] Machan R, Miszta A, Hermens W, Hof M (2010). Real-time monitoring of melittin-induced pore and tubule formation from supported lipid bilayers and its physiological relevance. Chem Phys Lipids.

[CR29] Madani F, Lindberg S, Langel Ü, Futaki S, Gräslund A (2011). Mechanisms of cellular uptake of cell-penetrating peptides. J Biophys.

[CR30] Marquette A, Lorber B, Bechinger B (2010). Reversible liposome association induced by LAH4: a peptide with potent antimicrobial and nucleic acid transfection activities. Biophys J.

[CR31] Mashaghi A, Swann M, Popplewell J, Textor M, Reimhult E (2008). Optical anisotropy of supported lipid structures probed by waveguide spectroscopy and its application to study of supported lipid bilayer formation kinetics. Anal Chem.

[CR32] Merz C, Knoll W, Textor M, Reimhult E (2008). Formation of supported bacterial lipid membrane mimics. Biointerphases.

[CR33] Nan YH, Park IS, Hahm KS, Shin SY (2011). Antimicrobial activity, bactericidal mechanism and LPS-neutralizing activity of the cell-penetrating peptide pVEC and its analogs. J Pept Sci.

[CR34] Nguyen LT, Haney EF, Vogel HJ (2011). The expanding scope of antimicrobial peptide structures and their modes of action. Trends Biotechnol.

[CR35] Niemelä PS, Ollila S, Hyvönen MT, Karttunen M, Vattulainen I (2007). Assessing the nature of lipid raft membranes. PLoS Comput Biol.

[CR36] Nordén B, Rodger A, Dafforn TR (2010). Linear dichroism and circular dichroism—a textbook on polarized-light spectroscopy.

[CR37] Oreopoulos J, Epand RF, Epand RM, Yip CM (2010). Peptide-induced domain formation in supported lipid bilayers: direct evidence by combined atomic force and polarized total internal reflection fluorescence microscopy. Biophys J.

[CR38] Persson D, Thorén PEG, Nordén B (2001). Penetratin-induced aggregation and subsequent dissociation of negatively charge phospholipid vesicles. FEBS Lett.

[CR39] Piantavigna S, McCubbin GA, Boehnk S, Grahamb B, Spiccia L, Martin LL (2011). A mechanistic investigation of cell-penetrating Tat peptides with supported lipid membranes. Biochim Biophys Acta.

[CR40] Raghuraman H, Chattopadhyay A (2004). Interaction of melittin with membrane cholesterol: a fluorescence approach. Biophys J.

[CR41] Richter RP, Berat R, Brisson AR (2006). Formation of solid-supported lipid bilayers: an integrated view. Langmuir.

[CR42] Rodahl M, Höök F, Krozer A, Brzezinski P, Kasemo B (1995). Quartz-crystal microbalance setup for frequency and Q-factor measurements in gaseous and liquid environments. Rev Sci Instrum.

[CR43] Rodrigues M, Santos A, de la Torre BG, Radis-Baptista G, Andreu D, Santos NC (2012). Molecular characterization of the interaction of crotamine-derived nucleolar targeting peptides with lipid membranes. Biochimica Et Biophysica Acta-Biomembranes.

[CR44] Róg T, Pasenkiewicz-Gierula M, Vattulainen I, Karttunen M (2009). Ordering effects of cholesterol and its analogues. Biochimica Et Biophysica Acta-Biomembranes.

[CR45] Runnels LW, Scarlata SF (1995). Theory and application of fluorescence homotransfer to melittin oligomerization. Biophys J.

[CR46] Rydberg HA, Carlsson N, Nordén B (2012). Membrane interaction and secondary structure of de novo designed arginine-and tryptophan peptides with dual function. Biochem Biophys Res Commun.

[CR47] Rydberg HA, Matson M, Åmand HL, Esbjörner EK, Nordén B (2012). Effects of tryptophan content and backbone spacing on the uptake efficiency of cell-penetrating peptides. Biochemistry.

[CR48] Rydell GE, Dahlin AB, Höök F, Larson G (2009). QCM-D studies of human norovirus VLPs binding to glycosphingolipids in supported lipid bilayers reveal strain-specific characteristics. Glycobiology.

[CR49] Sauerbrey G (1959). Verwendung von Schwingquarzen zur Wägung dünner Schichten und zur Mikrowägung. Zeitschrift Für Physik.

[CR50] Shaw JE, Epand RF, Hsu JCY, Mo GCH, Epand RM, Yip CM (2008). Cationic peptide-induced remodelling of model membranes: direct visualization by in situ atomic force microscopy. J Struct Biol.

[CR51] Splith K, Neundorf I (2011). Antimicrobial peptides with cell-penetrating peptide properties and vice versa. Eur Biophys J Biophys Lett.

[CR52] Staubitz P, Peschel A, Nieuwenhuizen WF, Otto M, Gotz F, Jung G, Jack RW (2001). Structure-function relationships in the tryptophan-rich, antimicrobial peptide indolicidin. J Pept Sci.

[CR53] Strömstedt AA, Ringstad L, Schmidtchen A, Malmsten M (2010). Interaction between amphiphilic peptides and phospholipid membranes. Curr Opin Colloid Interface Sci.

[CR54] Wadhwani P, Reichert J, Burck J, Ulrich AS (2011). Antimicrobial and cell-penetrating peptides induce lipid vesicle fusion by folding and aggregation. Eur Biophys J Biophys Lett.

[CR55] Wang Q, Hong GY, Johnson GR, Pachter R, Cheung MS (2010). Biophysical properties of membrane-active peptides based on micelle modeling: a case study of cell-penetrating and antimicrobial peptides. J Phys Chem B.

[CR56] Yeaman MR, Yount NY (2003). Mechanisms of antimicrobial peptide action and resistance. Pharmacol Rev.

