# Supplementary material for: Peptide-membrane interactions of arginine-tryptophan peptides probed using quartz crystal microbalance with dissipation monitoring
Source: Eur Biophys J. 2014 Apr 18;43(6):241–53. doi: 10.1007/s00249-014-0958-9 (PMC4053608; doi:10.1007/s00249-014-0958-9)
Supplement: Supplementary file 1 — Supplementary material 1 (DOCX 280 kb) [file 249_2014_958_MOESM1_ESM.docx]

# Peptide-membrane interactions of arginine-tryptophan peptides probed using quartz crystal microbalance with dissipation monitoring

Hanna A. Rydberg^1^, Angelika Kunze^2^, Nils Carlsson^1^, Noomi Altgärde^2^, Sofia Svedhem^2^ and Bengt Nordén^1^

^1^Dep. of Chemical and Biological Engineering, Chalmers University of Technology, Kemivägen 10, 412 96 Gothenburg, Sweden

^2^Dep. of Applied Physics, Chalmers University of Technology, Fysikgränd 3, 412 96 Gothenburg, Sweden

**Supplementary Information**

**The Supplementary Information includes:**

S1 Liposome aggregation, intensity profiles (figure)

S2 Tabled values of average frequency and dissipations shifts, and mass deposition

S3 Representative Δf–ΔD plots for the interaction of the RWpeptides with POPC/POPG and POPC/POPG/cholesterol membranes

S4 Calculation of amount of peptide binding per cm^2^ from carboxyfluorescein self-quenching experiments

S5 Peptide secondary structure when binding to membranes of different compositions (figure)

S6 Addition of liposomes to peptide treated POPC/POPG membrane monitored using QCM‑D

S7 References

**S1 Peptide-induced aggregation of liposomes**

**
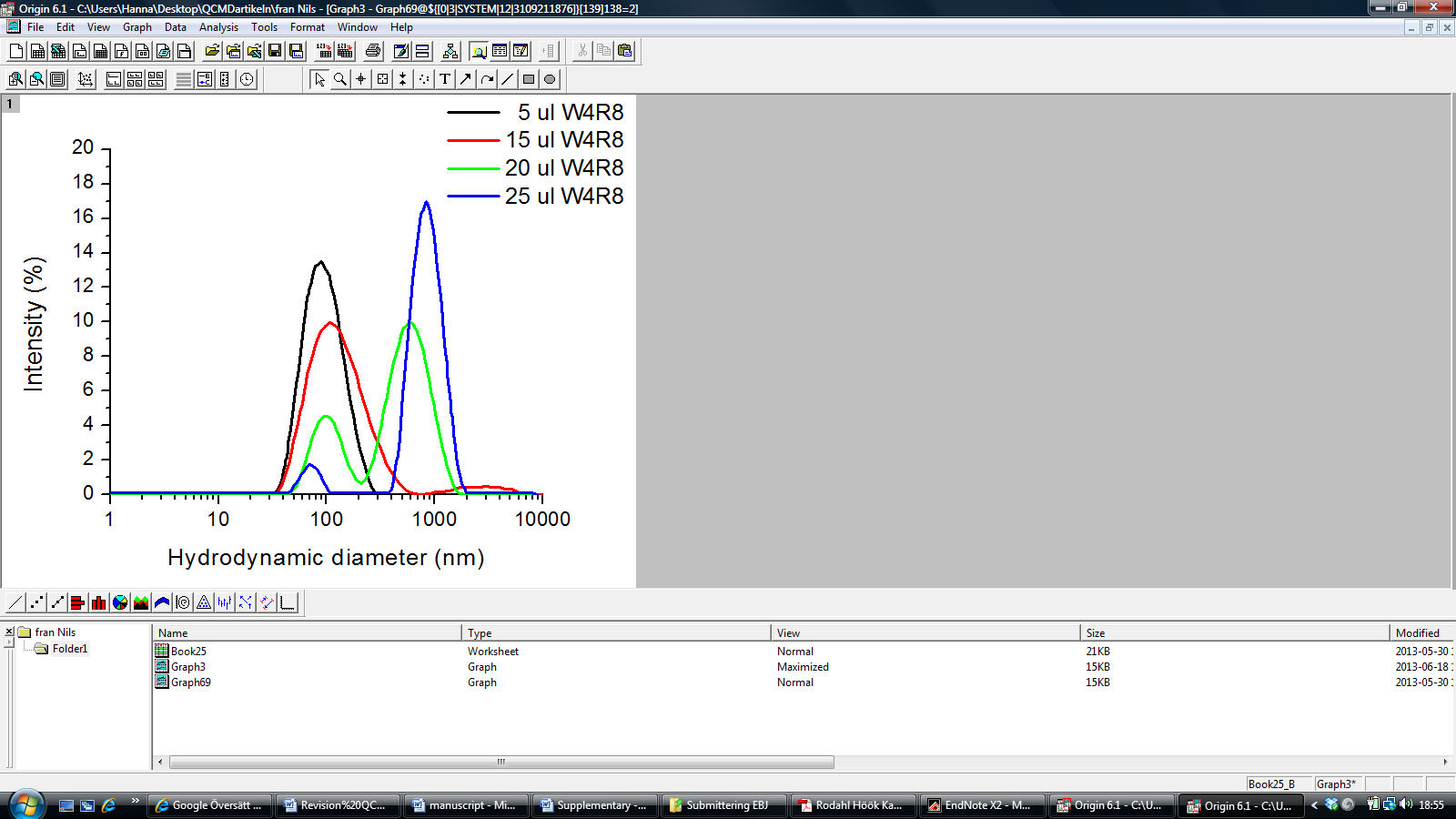
**

**Fig. S1** DLS intensity profiles of the change in hydrodynamic diameter of the peptide-liposome aggregates after addition of 5 µl (black), 15 µl (red), 20 µl (green) and 25 µl (blue) W_4_R_8_ (100 µM) respectively to 46 µM POPC/POPG liposomes in solution.

**S2 Average QCM-D frequency and dissipation shifts obtained upon binding of RW-peptides to four different lipid membranes**

**Table S1** Averages and standard deviations of QCM-D frequency and dissipation shifts (*Δf* and *ΔD*), compared with the initial values, at around 15 min after peptide addition (when maximum shifts in frequency are seen) and at around 80 min after peptide addition. The values are averages of 3 to 4 separate measurements. The calculated average values from *Δf_15min_* of mass deposition of peptide for each of the lipid membranes are also presented (values taking into account that about 50% of the frequency shift is due to water (Edvardsson et al. 2009), and assuming that the Sauerbrey equation is valid).

|  | | *Δf*_15min_ (Hz) | *Δf*_80min_ (Hz) | *ΔD*_15min_ (10^-6^) | *ΔD*_80min_ (10^-6^) | Mass deposition (ng/cm^2^) |
| --- | --- | --- | --- | --- | --- | --- |
| POPC | W_4_R_8_ | -0.5 ± 0.2 | 0.2 ± 0.6 | 0.1 ± 0.0 | 0.1 ± 0.1 | 4 |
|  | RWR | -0.2 ± 0.1 | 0.3 ± 0.2 | 0.1 ± 0.1 | 0.0 ± 0.1 | 2 |
|  | RWmix | -0.5 ± 0.1 | -0.5 ± 0.2 | 0.2 ± 0.1 | 0.2 ± 0.1 | 4 |
| PC/PG | W_4_R_8_ | -4.0 ± 0.2 | -2.7 ± 0.7 | 0.2 ± 0.0 | 0.1 ± 0.1 | 35 |
|  | RWR | -3.9 ± 0.7 | -1.5 ± 0.9 | 0.7 ± 0.0 | 0.6 ± 0.1 | 35 |
|  | RWmix | -3.4 ± 0.6 | -0.1 ± 0.9 | 1.0 ± 0.3 | 0.6 ± 0.5 | 30 |
| PC/PG/chol | W_4_R_8_ | -3.9 ± 1.1 | -2.9 ± 0.1 | 0.2 ± 0.1 | 0.1 ± 0.0 | 35 |
|  | RWR | -3.8 ± 0.5 | -2.7 ± 0.9 | 0.4 ± 0.2 | 0.3 ± 0.1 | 34 |
|  | RWmix | -3.4 ± 0.3 | -1.5 ± 1.2 | 0.6 ± 0.1 | 1.1 ± 0.1 | 30 |
| PC/Lactosyl PE | W_4_R_8_ | -0.7 ± 0.1 | 0.0 ± 0.1 | 0.1 ± 0.0 | 0.1 ± 0.1 | 6 |
|  | RWR | -1.2 ± 0.5 | -0.6 ± 0.4 | 0.2 ± 0.0 | 0.2 ± 0.0 | 11 |
|  | RWmix | -1.1 ± 0.2 | -0.6 ± 0.4 | 0.2 ± 0.0 | 0.2 ± 0.0 | 10 |

**S3 Representative Δf–ΔD plots for the interaction of the RW-peptides with POPC/POPG and POPC/POPG/cholesterol membranes**

**Fig. S2.** Representative Δf–ΔD plots for the interaction of W_4_R_8_ (light grey), RWR (grey) and RWmix (dark grey) with POPC/POPG (A) and POPC/POPG/cholesterol (B). The coordinate (0,0) corresponds to the time when the peptide is added to the membrane. Time increases along the trace and the last point in the trace corresponds to 80 min of peptide incubation. For the POPC/POPG membrane (left), RWR and RWmix show similar pattern, whereas the dissipation respons is very low and rather constant for W_4_R_8_ rendering a flat Δf-ΔD plot. When cholesterol is introduced to the membrane (right), RWmix shows a response evidently different to those of the other two peptides, which show similar pattern.

**S4 Calculation of the amount of peptide binding per cm^2^ (surface concentration) from self-quenching experiments**

Assuming that the liposomes are unilamellar (which they are supposed to be using the method described), the number of liposomes in the suspension for which a minimum in fluorescence could be observed was calculated as follows:

The number of lipid molecules in a liposome:

$$N=\frac{\left[ 4\pi\left( \frac{d}{2} \right)^{2}+4\pi\left[ \frac{d}{2}-h \right]^{2} \right]}{a}$$

Where, the surface area of a sphere: $A_{surface}=4\pi\left( \frac{d}{2} \right)^{2}$ , d = diameter of liposome which was 140 nm in this experiment, h= thickness of lipid bilayer which is around 5 nm and a= area of one lipid molecule head which around 0.7 nm^2^. The area of the head of POPC is 0.74 (outer leaflet) and 0.61 (inner leaflet) (Huang and Mason, 1978) but the liposomes used also contained 20 mole% POPG, and therefore an estimated average value of the lipid head group was used.

$N=\frac{\left[ 4\pi\left( 70 \right)^{2}+4\pi\left[ 65 \right]^{2} \right]}{0.7}=163812$ lipid molecules per liposome

The number of liposomes in the suspension: $N_{lipo}=\frac{n_{lipo}\times N_{A}}{N}$ , where n_lipo_ is the amount (in moles) of lipids in the suspension and N_A_ is Avogadros number which is 6.022∙10^23^. n_lipo_ used in this experiment was the lipid amount for where a minimum in the fluorescence intensity was monitored. For W_4_R_8_ it was 60 nmol, for RWR 100 nmol and for RWmix 80 nmol. The number of liposomes thus became, for W_4_R_8_ 2.2∙10^11^, for RWR 3.7∙10^11^ and for RWmix 2.9∙10^11^.

The total surface area of liposomes was calculated using $A_{surface total}=4\pi\left( \frac{d}{2} \right)^{2}\times N_{lipo}$, rendering areas of 135 cm^2^ for W_4_R_8_, 228 cm^2^ for RWR and 179 cm^2^ for RWmix.

The number of fluorescently labelled peptide molecules was calculated from the peptide concentration to, 1.4∙10^15^ for W_4_R_8_, 1.8∙10^15^ for RWR and 1.7∙10^15^ for RWmix.

The number of peptide molecules per cm^2^ thus become, for W_4_R_8_ 10.0∙10^12^, for RWR 7.9∙10^12^ and for RWmix 9.5∙10^12^. Or converted to mass per cm^2^: 35 ng/cm^2^ for W_4_R_8_, 26 ng/cm^2^ for RWR and 32 ng/cm^2^ for RWmix.

**S5 Peptide secondary structure when binding to membranes of different compositions**

**Fig. S3** CD assessment of secondary structure of RW peptides upon binding to POPC/POPG (black) and POPC/POPG/cholesterol (grey) liposomes respectively. The peptide concentration was 5μM and the lipid concentration was 0.5 mM. RWmix adopts a structure similar to that of an α-helix when interacting with liposomes of either composition, with a maximum below 200 nm and minima at around 205 and 222 nm. The other two RW-peptides show disordered (random coil) structures when binding to both membranes. RWR, however, shows a conspicuous negative peak at around 228 nm for both membranes, probably the result of interactions between adjacent tryptophan residues.

**S6 Addition of liposomes to peptide treated POPC/POPG membrane monitored using QCM-D**


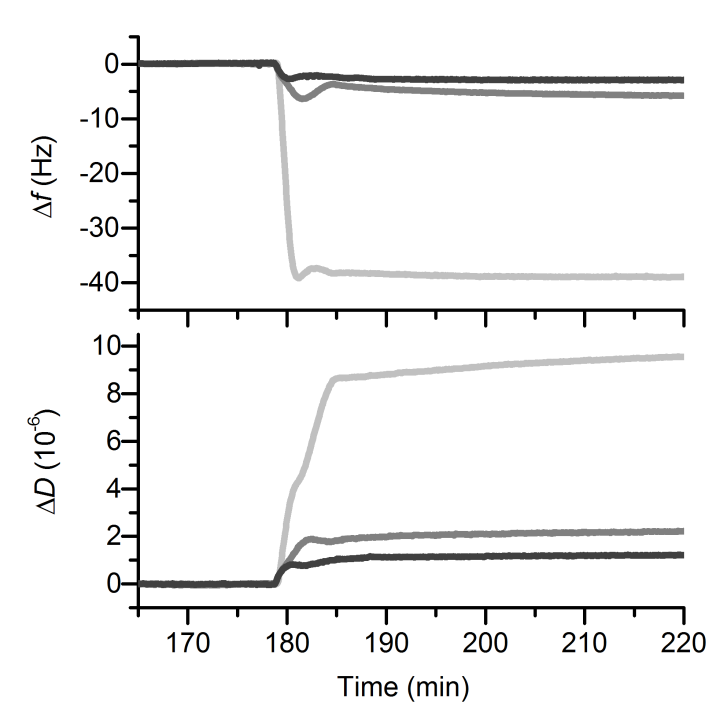


**Fig. S4** Frequency and dissipation shifts for addition of POPC/POPG liposomes to POPC/POPG membranes after treatment with peptide and wash with PBS. A flow of PBS is followed by addition of liposomes at around 180 min. Light grey curves represent membrane treated with W_4_R_8_, grey curves represent RWR and dark grey represent membrane treated with RWmix. All curves are normalized with respect to the *Δf* and *ΔD* values after wash with PBS. As can be seen by the decrease in frequency and increase in dissipation, liposomes are binding to membranes treated with all three peptides, i.e. there are still peptide present at all three surfaces since liposomes do not bind to untreated membranes. However, the liposome binding is higher for the W_4_R_8_-treated membrane, which may be a result of the more pronounced ability of this peptide to aggregate liposomes compared with the other two peptides. The difference in liposomes binding may also be related to differences in peptide concentration on the surface after wash with PBS.

**S7 References**

Edvardsson M, Svedhem S, Wang G, Richter R, Rodahl M, Kasemo B (2009) QCM-D and Reflectometry Instrument: Applications to Supported Lipid Structures and Their Biomolecular Interactions. Analytical Chemistry 81:349-361
